# Cost-Effective Leachate Treatment and Resource Recovery in Hazardous Waste Landfills through Pipe Freeze Crystallization

**DOI:** 10.1007/s40710-025-00757-3

**Published:** 2025-03-24

**Authors:** Kagiso S. More, Johannes P. Maree, Mlungisi Mahlangu

**Affiliations:** Institute for Nanotechnology and Water Sustainability, College of Science, Engineering and Technology, University of South Africa, Private Bag X6, Science Campus, Florida, 1709 Johannesburg South Africa

**Keywords:** Sustainable wastewater treatment, Energy-efficient desalination, Resource recovery technology, Zero-waste wastewater treatment, Industrial effluent treatment

## Abstract

This study aimed to develop a practical, economically viable solution for treating hazardous landfill leachate using Pipe Freeze Crystallization (PFC) technology. The objective was to concentrate and solidify leachate from an effluent treatment plant processing approximately 8750 m^3^ annually, achieving resource recovery and environmental compliance. A 300 L h^-1^ cooling demonstration plant was designed and implemented, incorporating a chiller, a secondary refrigerant mixture (40% ethylene glycol and 60% water), a clarifier, a reactor, and pumps. Μodelling with OLI software estimated recovery rates for salt and ice, providing a basis for operational adjustments. Leachate samples (2000 L) and concentrate (1000 L) were processed to evaluate the plant’s performance in recovering clean water and Na_2_SO_4_. Experimental results confirmed the model predictions, with 302 L of concentrate yielding 102.9 kg of Na_2_SO_4_ over 6 h and 273 L of leachate producing 118.7 kg of high-purity ice over 5.5 h. The energy consumption was measured at 171 kWh t^-1^ of ice, aligning with theoretical predictions for a coefficient of performance of 1. These results validate the efficiency and feasibility of PFC in resource recovery. This study highlights the importance of PFC as a low-cost, energy-efficient technology for hazardous leachate treatment. Its scalability and ability to recover valuable resources such as Na_2_SO_4_ and clean water present a sustainable alternative to conventional methods, contributing to zero-waste management goals in waste treatment practices.

**Supplementary Information:**

The online version contains supplementary material available at 10.1007/s40710-025-00757-3.

## Highlights

• Pipe Freeze Crystallization recovers Na_2_SO_4_, NaCl, and clean water from leachate.

• The process is 40% more cost-effective than evaporative-based methods.

• It consists a scalable technology suitable for industrial applications.

• Enables recovery of high-purity salts and water, supporting circular economy goals.

**Supplementary Information:**

The online version contains supplementary material available at 10.1007/s40710-025-00757-3.

## Introduction

Leachate management is a challenge in the operation of hazardous waste landfills. As rainwater and other liquids percolate through the waste materials, they dissolve and carry away various contaminants, forming leachate, a highly concentrated, hazardous liquid that poses serious environmental risks (Pavelka et al. 1993; Teng et al. 2021). Effective treatment of leachate is important to prevent contamination of groundwater and soil, as well as to comply with the environmental regulations (e.g. Department of Environmental Affairs 2013). Traditional methods of leachate treatment, such as biological treatment (Hussain et al. 2021; Kanaujiya et al. 2019; Machineni 2019), chemical precipitation (e.g. Zhang et al. 2009) and membrane filtration (e.g. Goh and Ismail 2018; Greenlee et al. 2009), have limitations in terms of efficiency, cost and the ability to handle highly concentrated waste streams. Recent advancements in leachate treatment explore innovative approaches to address these challenges. For example, microbial fuel cells have shown good performance in treating volatile fatty acid rich leachate while simultaneously generating electricity (Gurjar and Behera 2022). Other emerging techniques, such as hybrid sono-electrocoagulation (Asaithambi and Govindarajan 2021) and adsorption using wood-derived biochar (Zand and Abyaneh 2020), offer potential solutions for improving the efficiency of leachate management. Other methods include anaerobic reactors with constructed wetlands for contaminant reduction (Galindo Montero et al. 2019), intermittent aeration for nutrient removal (Melidis 2014), and the Fenton process to improve leachate biodegradability (Bernardo-Bricker et al. 2014).

In recent years, freeze crystallization has emerged as one of the useful alternatives for the treatment of complex waste streams such as leachate, reverse osmosis brine and seawater brine (Kolliopoulos et al. 2018). This process uses the principle of lowering the temperature to separate contaminants from water, producing clean ice and recovering salts (El Kadi and Janajreh 2017; Maree 2018; More et al. 2024; Randall and Nathoo 2015). Freeze crystallization is particularly advantageous because it can handle high concentrations of salts and other contaminants, has relatively low energy requirements, does not require the usage of chemicals, and can recover valuable resources from the waste streams (e.g. Mtombeni et al. 2013; Randall and Nathoo 2018).

**Fig. 1:**
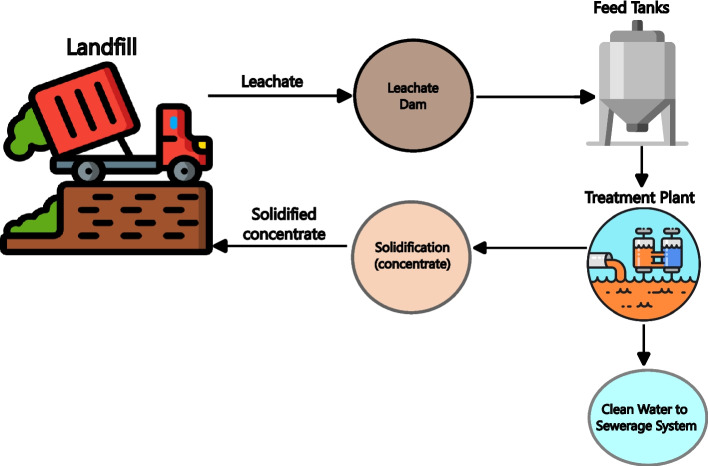
Current leachate treatment process at the waste management company in South Africa

This study is based on leachate produced by a waste management company in South Africa which operates a liquid, solid hazardous and non-hazardous waste (municipal solid waste and commercial and industrial waste) treatment plant and faces the challenge of managing and treating the large volumes of leachate produced. The current methods, (distillation; evaporation ponds; reverse osmosis; Panagopoulos et al. 2019; Sherwood et al. 1967), are not only costly but also inadequate in handling the increasing volume and concentration of leachate. Figure 1 illustrates the leachate treatment process at a South African waste management company, where leachate moves from the landfill to a leachate dam, feed tanks, and a treatment plant, with treated water discharged and solidified residue returned to the landfill to be treated again. This study aims to address this issue by developing a practical, feasible and economically viable technology to solidify the concentrated leachate from the effluent treatment plant. The goal is to treat approximately 8750 m^3^ of leachate annually, reducing environmental impact and operational costs.

The primary objective of this study is to develop and evaluate a demonstration plant for pipe freeze crystallization (PFC) of leachate, with the following specific aims: i) design and construct a 300 L h^−1^ cooling demonstration plant equipped with the necessary components for freeze crystallization (Pipe Freeze Crystallization; Maree 2018), including chiller, secondary refrigerant mixture, clarifier, reactor and pumps; ii) assess the performance of the demonstration plant in terms of its ability to recover clean water and salts from leachate, including measuring the quantities of ice and Na_2_SO_4_ produced, and comparing these results with predictive models; iii) conduct a comprehensive cost analysis of the freeze crystallization process, comparing it to traditional leachate treatment methods, including evaluating the energy consumption and overall economic viability of the proposed solution; and iv) evaluate the environmental benefits of the freeze crystallization process, particularly in terms of reducing brine and recovering valuable resources. Furthermore, this technique’s goal is to reduce brine concentration, so that the discharged wastewater into water bodies (e.g., rivers) has acceptable brine concentration. In terms of competition, this technique does not necessarily compete with or seek to replace other wastewater treatment methods. In fact, it competes with the evaporative-based methods which are mainly used for treatment and management of highly saline streams. Evaporation ponds are an environmental hazard as they contaminate soil and groundwater (Naicker et al. 2003; Panagopoulos et al. 2019), while distillation uses a lot of energy for brine treatment (Soliman et al. 2021).

Despite advancements in freeze crystallization technology, current methods rely heavily on mechanical scrapers to remove ice from cold surfaces, presenting operational and maintenance challenges (Samah et al. 2023). Scrapers often lead to equipment wear and increased operational costs, while the ice produced typically contains impurities, reducing its utility as clean water when melted (Motsepe 2021). In contrast, the PFC method developed in this study eliminates the need for scrapers, allowing ice to form as a slurry floating in the clarifier alongside brine (Maree 2018; More et al. 2024). This innovative approach not only simplifies the process but also enhances the purity of the recovered ice. Once melted, this pure ice provides clean water that can be reused, contributing to sustainable water management practices in hazardous waste treatment.

The predominant methods for brine treatment, such as distillation and evaporation ponds, have many limitations. Distillation, though effective, demands high energy, consuming approximately 2,200 kJ kg^−1^ of treated water compared to just 330 kJ kg^−1^ for PFC, making the PFC technology more energy-efficient (Soliman et al. 2021). Evaporation ponds, on the other hand, pose severe environmental risks as metals and salts can infiltrate the soil, leading to groundwater contamination (Amoatey et al. 2021; Cipolletta et al. 2021; Mapanda et al. 2007). In addressing these gaps, this study positions PFC technology as a cost-effective, environmentally friendly, and scalable alternative for treating hazardous landfill leachates while recovering valuable resources.

This study is important as it addresses environmental problems by providing an effective solution for treating hazardous leachate. The successful implementation of freeze crystallization technology can reduce the environmental impact of hazardous waste landfills. Furthermore, this study offers a cost-effective alternative to traditional treatment methods, potentially saving substantial amounts of money in waste management operations. In addition, as the proposed method in this study recovers valuable resources such as clean water and salts, it adds an economic incentive for its adoption, making it an attractive option for waste management companies (Maree 2018; More et al. 2024; Mtombeni et al. 2013; Zikalala et al. 2017). The novelty of PFC lies in its innovative approach to ice and salt recovery, which eliminates the reliance on mechanical scrapers typically used in conventional freeze crystallization systems. Instead, ice forms as a slurry in the clarifier, allowing for seamless separation and recovery, resulting in higher-purity ice that, when melted, yields reusable clean water.

## Theoretical Framework and Context

### Leachate Characteristics and Challenges

The leachate is a complex and highly variable liquid waste that results from the percolation of water through landfills containing hazardous materials. Its composition depends on the types of waste present, the age of landfill and the local climatic conditions (Wiszniowski et al. 2006). Typically, leachate contains a mix of organic and inorganic compounds, metals and other hazardous substances. The high concentration of these contaminants makes leachate management a serious environmental challenge. If not properly treated, leachate can contaminate groundwater and surface water, threating public health and aquatic life (Kjeldsen et al. 2002). Leachate management requires careful monitoring and effective treatment strategies to mitigate these risks and ensure environmental safety.

One of the major challenges in leachate management is its heterogeneity. The composition of leachate can vary largely not only between different landfill sites but also within the same site over time. This variability complicates the design and implementation of treatment systems, as they must be flexible enough to handle changing contaminant loads. Furthermore, the presence of various metals and persistent organic pollutants adds another layer of complexity. These substances are often resistant to conventional treatment methods, necessitating the development of more versatile approaches (Renou et al. 2008).

### Current Leachate Treatment Methods

Evaporative-based methods, such as distillation and evaporation ponds, are commonly used for treating highly saline leachate due to their ability to concentrate contaminants effectively (Amoatey et al. 2021; Nannarone et al. 2017; Panagopoulos et al. 2019; Roychoudhury and Petersen 2014; Soliman et al. 2021). Distillation, which involves heating leachate to separate water from dissolved solids, is highly energy-intensive (Soliman et al. 2021). This significant energy demand translates into high operational costs, making distillation economically unsustainable for large-scale applications. Additionally, the process is heavily reliant on consistent energy inputs, which can pose challenges in regions with limited energy infrastructure. Evaporation ponds, designed to use natural solar energy for water evaporation, are comparatively simpler, but present substantial environmental risks. Metals, salts, and other contaminants in the leachate can infiltrate the soil and groundwater, posing long-term environmental risks (Amoatey et al. 2021; Panagopoulos et al. 2019). In addition, evaporation ponds require large land areas, making them impractical for urban or space-constrained settings, and their dependence on climatic conditions can limit their effectiveness in regions with lower evaporation rates. These limitations highlight the need for alternative, energy-efficient, and environmentally sustainable solutions, such as freeze crystallization.

Other techniques such as the biological treatment methods, including aerobic and anaerobic processes, rely on microbial activity to break down organic contaminants (Dutta and Bhattacharjee 2022; Hussain et al. 2021; Wainaina et al. 2020). While effective under certain conditions, these methods can be limited by the presence of toxic substances and the need for extensive post-treatment to handle residuals (Zieliński et al. 2022). For example, the nitrification–denitrification process, widely used for ammonia removal, is sensitive to fluctuations in temperature and pH, which can affect microbial activity and overall treatment efficiency (Ahn 2006). Chemical precipitation involves the addition of chemicals to induce the formation of insoluble precipitates, which can then be removed by sedimentation or filtration. This method is particularly effective for removing toxic metals and phosphates (Chen et al. 2022; Kang et al. 2017; Yigit and Mazlum 2007). However, the generation of large volumes of sludge presents a disposal challenge and increases the overall cost of the treatment process. In addition, the effectiveness of chemical precipitation can be influenced by the presence of competing ions and the specific chemistry of the leachate (Fu and Wang 2011). Membrane filtration technologies, such as reverse osmosis, nanofiltration and ultrafiltration, offer high removal efficiencies for a wide range of contaminants. These methods operate by applying pressure to force water through a semi-permeable membrane, leaving contaminants behind. While effective, membrane technologies are often associated with high capital and operational costs, primarily due to energy requirements and the need for frequent membrane cleaning and replacement to address fouling issues (Mahlangu et al. 2023; Trebouet et al. 2001).

### Freeze Crystallization in Waste Management

Freeze crystallization is an innovative treatment process that involves lowering the temperature of leachate to form ice crystals and a concentrated brine. The ice crystals, which are relatively pure, can be separated from the brine, resulting in the recovery of clean water and the concentration of contaminants in the brine. This method is particularly suitable for leachate treatment due to its ability to handle high concentrations of salts and other contaminants without the need for extensive chemical additives (Lewis et al. 2008; Randall and Nathoo 2018).

Freeze crystallization offers several advantages over traditional methods. It has lower energy requirements because the latent heat of fusion is lower than the latent heat of vaporization used in evaporation processes (Randall and Nathoo 2018). In addition, it can operate effectively at low temperatures, reducing the risk of volatising hazardous compounds. Furthermore, freeze crystallization allows for the recovery of valuable resources such as Na_2_SO_4_, which can be reused in various industrial applications, or be converted to Na_2_CO_3_ through thermal treatment which is an economically valuable product as it is used in the manufacturing of soaps and detergents.

The process of freeze crystallization can be divided into two main stages, i.e., nucleation and crystal growth. Nucleation is the initial phase where small ice crystals form, while crystal growth involves the enlargement of these crystals as more water molecules join the lattice structure (Myerson and Ginde 2002). Contaminants are typically excluded from the ice lattice during crystal formation, leading to the production of relatively pure ice. The remaining liquid becomes concentrated in contaminants, which can then be separated and treated further if necessary (El Kadi and Janajreh 2017; Janajreh et al. 2023).

One of the key factors influencing the efficiency of freeze crystallization is the rate of cooling. Rapid cooling can lead to the formation of small ice crystals of higher purity, while slower cooling rates may result in larger crystals with slightly lower purity. The choice of cooling rate depends on the specific treatment goals and the nature of the leachate being treated. In addition, the use of secondary refrigerants, such as ethylene glycol and water mixture (as used in this study), can enhance the efficiency of the process by providing better heat transfer and control over the freezing conditions (e.g. Zikalala et al. 2017).

## Materials and Methods

### Sample Collection and Water Quality

Two types of samples were collected: i) 1000 L of concentrate from the evaporation unit; and ii) 2000 L of raw leachate (feed to the evaporator). Table 1 presents key parameters relevant to this study, while the complete chemical composition is provided in Supplementary Material (SM) Table SM1. Chemical analysis of the samples revealed total dissolved solids (TDS) concentrations of 220,201 mg L^−1^ for the concentrate and 50,005 mg L^−1^ for the leachate, closely aligning with the company-provided values of 270,000 mg L^−1^ and 50,000 mg L^−1^, respectively.

**Table 1:** Chemical composition of samples collected at the waste management company on 14 February 2024; COD: chemical oxygen demand, TDS: total dissolved solids

Parameter	Sample A: Evaporation Concentrate	Sample B: Leachate
pH	6.69	8.64
COD, mg L^−1^ O_2_	39,900	10,400
Total Conductivity, mS m^−1^	11,900	3,810
Cl^−^, mg L^−1^	25,953	8,238
NO_3_^−^, mg L^−1^	55.60	6.27
PO_4_^3−^, mg L^−1^	47.51	22.34
SO_4_^2−^, mg L^−1^	121,700	16,330
TDS, mg L^−1^	220,201	50,005

The sampling procedure followed industry-standard protocols to ensure representative and uncontaminated samples. At the landfill site, the concentrate and leachate were collected directly from the designated collection points into large, clean, and sealed high-density polyethylene drums to prevent contamination or loss of volatile compounds. The samples were homogenised by stirring before transfer to ensure uniformity and accuracy in subsequent analysis. Each drum was labelled with sample type, date, and source details.

The drums were securely loaded onto trucks equipped with safety measures to prevent spillage or exposure during transportation to the PFC plant. Upon arrival, the samples were stored in a temperature-controlled environment to preserve their chemical integrity until analysis and treatment experiments commenced. This rigorous procedure ensured that the samples were representative of the leachate and concentrate streams, allowing for reliable assessment of the freeze crystallization process.

### Design and Development of the Demonstration Plant

#### Equipment and Setup

The PFC demonstration plant used for the treatment of leachate from the hazardous waste landfill consists of several key components designed to facilitate the efficient separation and recovery of clean water and salts. The setup includes an 18 kW chiller equipped with a primary refrigerant, which is essential for reducing the temperature of the secondary refrigerant and initiating the crystallization process. This chiller is important for maintaining the low temperatures necessary for effective freeze crystallization. Different chiller set-point temperatures, including –8 °C, –10 °C, –16 °C, –20 °C and –21 °C, were examined in this study.

Central to the system is the pipe heat exchanger, where the primary refrigerant cools a secondary refrigerant mixture composed of 40% ethylene glycol and 60% water. This mixture is selected for its effective thermal properties and ability to maintain the low temperatures required for the process. This mixture was tested through beaker studies using the water bath which cools down to –20 °C; the mixture was placed inside a beaker and this beaker inside the water bath and was monitored to determine the freezing point of the mixture. The mixture did not form any ice crystals during the experiments, which indicated that it is good for freeze crystallization studies. The heat exchanger plays an important role in transferring cold to the leachate, helping with the crystallization of salts and the formation of ice.

A 200 L and 1000 L clarifiers were used for the separation of salt, liquid and ice. The clarifiers ensure that the solidified ice and crystallized salts are efficiently separated from the liquid brine, which is important for the purity and quality of the recovered products. In addition, a filter with 1 mm pore spaces was used to separate the liquid from the ice, ensuring that the ice produced is as pure as possible and free from residual contaminants.

**Fig. 2:**
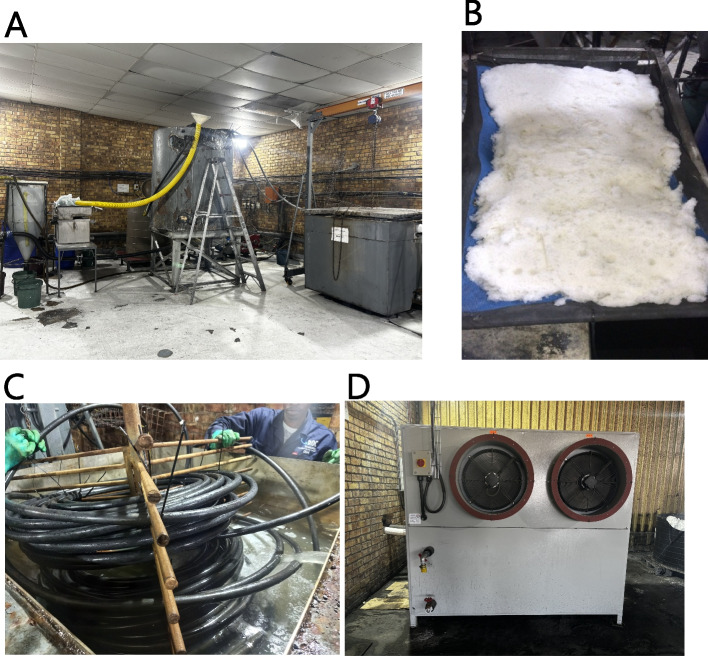
Freeze crystallization demonstration plant for the leachate treatment; **A** from left to right: 200 L clarifier, 1000 L clarifier (in the middle), and a cooler reactor; **B** recovered ice on a filter net with 1 mm pore spaces; **C** PVC pipe on a skeleton, arranged in a coil-like shape, immersed in a cooler reactor with secondary refrigerant (40% ethylene glycol in water); **D** 18 kW chiller (primary refrigerant)

The equipment setup, as shown in Fig. 2, includes the cooler reactor (heat exchanger), the clarifiers, chiller and the ice filter, providing a comprehensive system for the PFC process. This arrangement not only optimises the process but also demonstrates the practical application of this technology in managing leachate from hazardous waste landfills. The integration of these components ensures the efficient recovery of purified water and valuable salts, showcasing the potential of freeze crystallization as a cost-effective and environmentally friendly solution for leachate treatment.

#### Freeze Crystallization Process

**Fig. 3:**
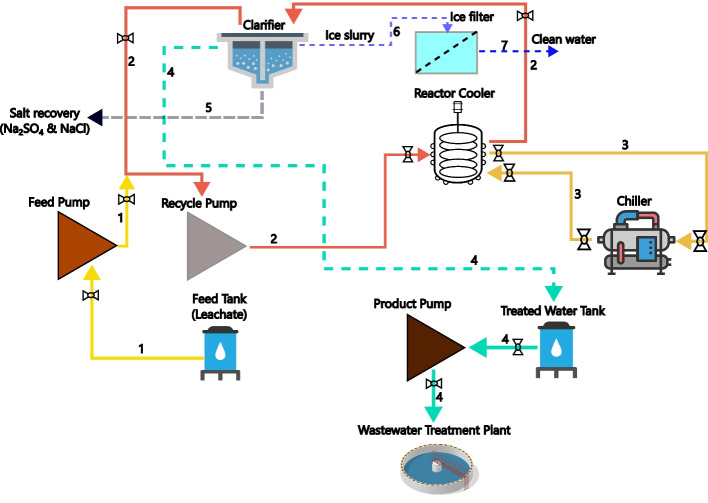
Schematic diagram of pipe freeze crystallization; 1: leachate pumped from the feed tank; 2: leachate circulation from the clarifier through the reactor cooler and back to the clarifier; 3: refrigerant circulates from the reactor cooler to the chiller and back to the reactor cooler; 4: product water recovery with less TDS; 5: Na_2_SO_4_.10H_2_O recovery; 6: ice slurry; 7: melted ice; dashed line: no pump involved; solid line: pump involved

The demonstration unit for PFC, capable of processing 300 L h^−1^, was designed to evaluate the scalability and practicality of the technology for industrial applications. This unit uses several key components (Fig. 3), each playing a specific role in the freeze crystallization process. The central element of the unit is an 18 kW chiller responsible for maintaining the low temperatures necessary for the crystallization of salts from the leachate. The chiller uses a primary refrigerant to achieve the required cooling capacity, ensuring efficient and consistent operation. Another key component is a pipe heat exchanger, which is also referred to as the cooler reactor, which transfers the cooling effect from the primary refrigerant to the secondary refrigerant. The secondary refrigerant, consisting of a 40% ethylene glycol solution in water, is cooled by the primary refrigerant within the heat exchanger. This setup enables the secondary refrigerant to reach the desired low temperatures essential for the freeze crystallization process. The choice of ethylene glycol in the secondary refrigerant mixture is due to its low freezing point and good thermal conductivity, supporting the performance of the system.

Following the heat exchanger, the leachate is recycled between the cooler reactor and the 1000 (and/or 200) L clarifier. The clarifier is designed for the separation of salt, liquid and ice. As the leachate passes through the cooler reactor, the lower temperatures induce the formation of ice crystals and the crystallization of salts, then these get deposited in the clarifier. The clarifier acts as a collection point of these solids while allowing the liquid brine to be separated and removed from the system. This separation is important for producing clean ice and recovering valuable salts from the leachate. In addition, the demonstration plant includes a filter designed for liquid and ice separation. After the initial separation in the clarifier, the mixture of liquid brine and ice passes through this filter. The filter removes any remaining liquid from the ice, ensuring that the recovered ice is pure. This step is important for producing good quality ice suitable for various applications and for maximising the recovery of clean water from the leachate.

The demonstration plant’s design and operation were planned to simulate real-world conditions and challenges encountered in industrial settings. The integration of the chiller, heat exchanger, clarifier and filter within the plant allows for a comprehensive evaluation of the freeze crystallization process. Processing leachate at a rate of 300 L h^−1^ provides important information into the scalability, efficiency and potential industrial implementation of freeze crystallization technology.

The experimental procedure was designed to thoroughly investigate the efficacy of freeze crystallization technology under different cooling conditions, specifically ambient cooling and chiller cooling/freezing. The experiments aimed to assess various parameters affecting the process, such as energy usage, ice purity and salt recovery, across different operational setups and conditions. Under ambient cooling, several key features were explored to understand the fundamental dynamics of the freeze crystallization process. The effect of water depth on ambient cooling was studied to determine how varying depths influenced the rate and efficiency of cooling. This aspect was important for optimising the initial stages of the process. In addition, the energy usage and associated costs for salt recovery (specifically Na_2_SO_4_) using freeze crystallization were analysed to evaluate the economic viability of the technology. Ice purity was an important focus, with investigations into the influence of feed rate, recycle rate, and the recirculation of ice crystals on the quality of the produced ice. The goal was to identify the optimal conditions that would maximise ice purity and ensure effective salt recovery from the brine solutions.

The chiller cooling/freezing experiments involved semi-batch runs to simulate a more controlled and scalable industrial application. Leachate was continuously recycled through the heat exchanger and a brine/ice filter. Product ice was continuously removed and replaced with feed water, repeating the process until the system volume had been treated. This repetitive approach allowed for a comprehensive analysis of the system’s performance and the quality of the recovered ice. Feed, ice, and brine samples were regularly analysed for Cl^−^, TDS, ice content, pH and conductivity to monitor the effectiveness of the crystallization process and the purity of the recovered water. Several parameters were investigated for their impact on ice purity under chiller cooling conditions. These included the type of salts present in the brine (Na_2_SO_4_), the temperature of the secondary refrigerant and the recycle flow rate. The temperature of the secondary refrigerant was varied to determine its effect on the freezing process and ice quality. The length and material of the pipes used in the system were also evaluated to identify the optimal configuration for efficient heat transfer and ice formation. Furthermore, the recycle flow rate was adjusted to understand its influence on the overall system performance and ice purity.

### Predictive Modelling with OLI Software

OLI software (Oli Systems Inc. 2021) was used in this study to help in predicting the solubility of salts and the formation of ice under different conditions, and in guiding the experimental setup and validate the results obtained from the demonstration plant. The OLI Systems software is a tool designed for water chemistry and electrolyte thermodynamics analysis, offering advanced capabilities for process simulation and prediction. At its core, the software operates on a rigorous thermodynamic framework, using models like the Mixed-Solvent Electrolyte model and the Aqueous Electrolyte model to simulate electrolyte behaviour in complex aqueous systems. These models predict properties such as solubility, speciation, phase equilibria, and reaction kinetics, providing insights into both liquid and solid phases under varying conditions.

The software allows for the input of detailed system parameters, including temperature, pressure, pH, and chemical composition, to simulate real-world conditions accurately. It generates comprehensive outputs, such as the concentrations of individual ions, precipitation of solid phases, and phase transitions. These outputs are invaluable for optimising chemical processes, designing treatment methods, and troubleshooting operational inefficiencies. OLI Systems also includes modules like the Stream Analyzer, which performs single-point or multi-point calculations for trend analysis, and provides results in formats for diverse applications. This feature enables users to evaluate the behaviour of complex mixtures, optimise operating conditions, and predict system performance with high precision.

When simulating the freeze crystallization process, it is possible to estimate the quantities of ice and salts that can be recovered, thereby helping in the design and optimisation of treatment plants. OLI simulation was used to predict the amount of salt and ice that will be produced when the samples are treated at the plant. The simulation was first conducted for the concentrate which showed the water quality (solids produced and compounds in solution) through the various stages of freeze crystallization (Table 2; full results on Table SM2).

**Table 2:** Predicted water quality through various stages of freeze crystallization using OLI software; sample: concentrate

Parameter	Feed	Cooling	Freeze 1	Freeze 2	Freeze 3
Water, kg h^−1^	1000	739.0	116.3	22.70	11.99
Ice recovery, %	–	–	64.60	9.400	1.100
Temperature, °C	25	0	−4	−21	−21
pH	7.570	7.930	7.220	6.670	6.710
Solids					
Ice, kg h^−1^	–	–	645.9	93.58	10.71
Na_2_SO_4_.10H_2_O, kg h^−1^	0.000	354.0	41.04	0.000	0.000
NaCl, kg h^−1^	–	–	0.000	25.23	2.940
Solution					
TDS, kg h^−1^	228.8	72.60	51.32	12.01	6.940
Cl^−^, kg h^−1^	25.95	25.90	25.84	5.330	3.380
Energy (cooling), J L^−1^ Feed	–	6,180	9,235	–	–
Energy (freezing), J L^−1^ Feed	–	–	–	31,163	3,566

The process to treat leachate was similar to the one used for the concentrate, and the process used to perform predictive analysis with the OLI software was also similar; however, the amounts of solids produced differed (Table 3; full results in Table SM3).

**Table 3:** Predicted water quality through various stages of freeze crystallization using OLI software; sample: leachate

Parameter	Feed	Cooling	Freeze 1	Freeze 2
Water, kg h^−1^	982.1	957.8	101.3	6.000
Ice recovery, %	–	–	856.5	95.31
Temperature, °C	25	0	−4	−21
pH	–	–	7.500	6.710
Solids				
Na_2_SO_4_.10H_2_O, kg h^−1^	0.000	43.42	71.99	1.020
Solution				
TDS, kg h^−1^	50.01	48.81	12.93	2.180
Cl^−^, kg h^−1^	7.000	7.000	7.000	1.220

## Results and Discussion

### Performance of the Demonstration Plant

#### Salt Recovery and Energy Usage

**Fig. 4:**
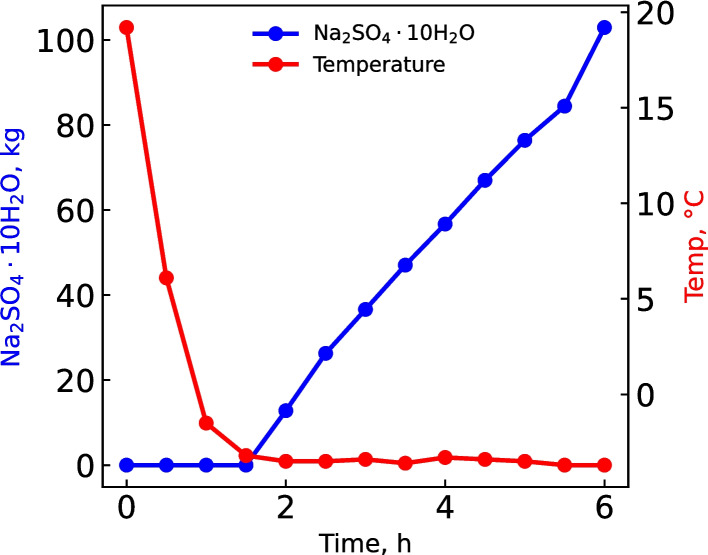
Na_2_SO_4_.10H_2_O production during cooling of the concentrate

The results predicted by OLI simulations were confirmed by actual runs on the 300 L h^−1^ demonstration plant. The salt production was monitored over time when 302 L of the concentrate was recycled. A mass of 102.9 kg salt (Na_2_SO_4_.10H_2_O) was recovered over a period of 6 h (Fig. 4), which amounts to 340 g L^−1^ as Na_2_SO_4_.10H_2_O. This corresponds well with the 354 g L^−1^ as predicted by OLI. Furthermore, the chemical composition of the brine was examined after 2, 3 and 4.5 h, and the energy usage was recorded over 6 h (Table 4).

**Table 4:** Energy usage and effect of time on the chemical composition of brine as salt is removed from the concentrate

Parameter	Time (h)				
	0.0	2.0	3.0	4.5	6.0
Volume, L	273	286	310	340	376
Temperature, °C	19.2	–3.50	–3.40	–3.40	–3.70
Na_2_SO_4_.10H_2_O, kg	0.00	12.8	36.6	67.0	103
Na_2_SO_4_.10H_2_O kg h^−1^	–	12.8	23.8	20.2	24.0
pH	–	6.98	8.1	6.72	–
TDS, g L^−1^	–	184	134	196	–
Cl^−^, g L^−1^	–	34.0	34.9	32.1	–
Energy, kWh	0.00	6.60	9.90	14.9	19.8
Energy usage, kWh t^−1^ salt	–	516	139	163	138

The actual recovery of 102.9 kg of Na_2_SO_4_.10H_2_O from the demonstration plant closely matched the OLI simulation predictions, indicating the effectiveness and accuracy of the process design. This close alignment between the predicted concentration of 353.9 g L^−1^ and the actual 339.8 g L^−1^ further validates the freeze crystallization approach. Energy usage data, collected alongside brine composition analyses over the 6-h operation, confirmed that the process is not only viable, but also energy efficient, highlighting its potential for industrial scale application.

**Fig. 5:**
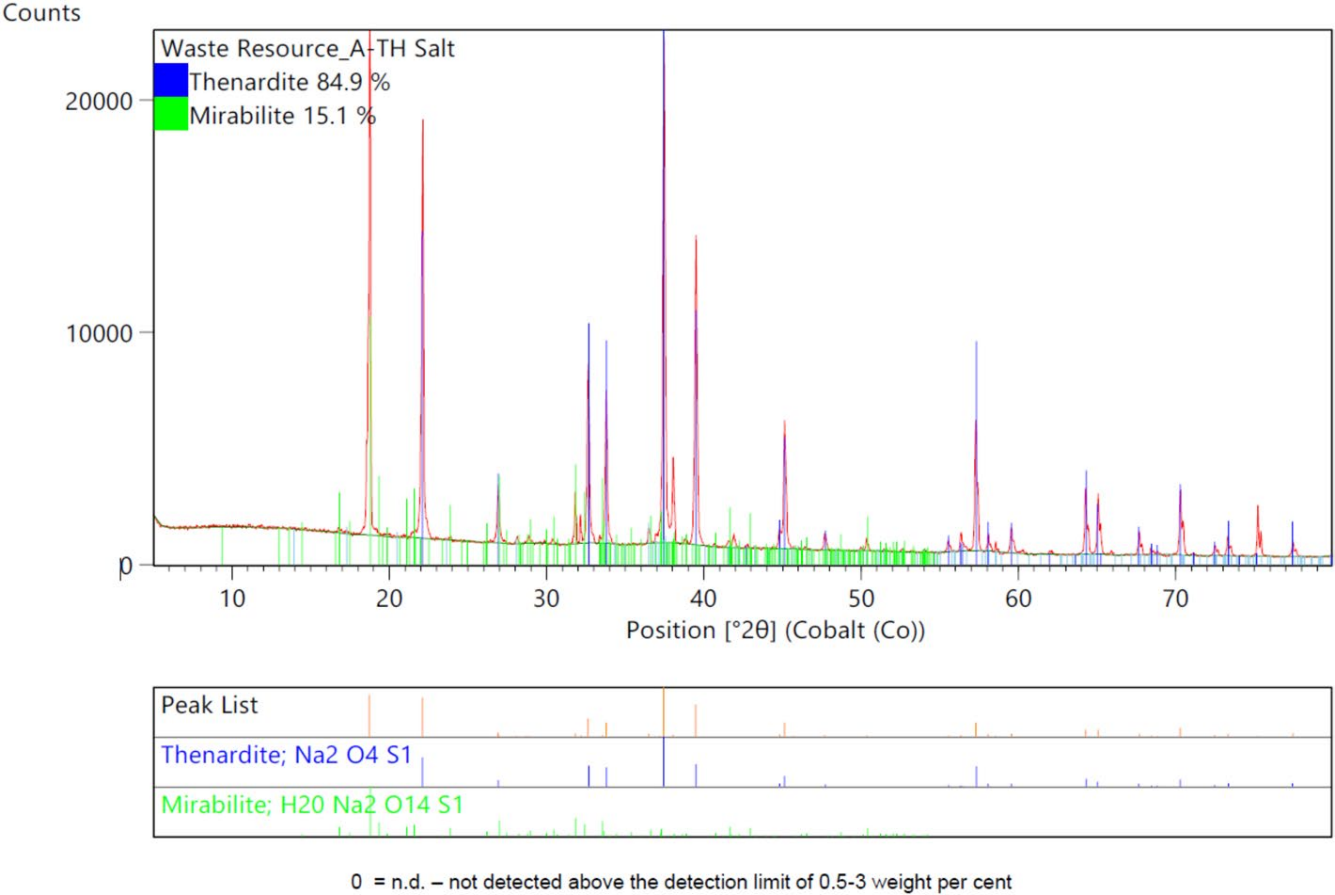
XRD analysis of the recovered Na_2_SO_4_ using the PFC technology

The purity of the recovered Na_2_SO_4_∙10H_2_O was evaluated using X-ray Diffraction (XRD) analysis. The slightly moist material obtained after the freeze crystallization process was prepared for analysis using a back-loading preparation method. Diffractograms were recorded using a Malvern Panalytical Aeris diffractometer equipped with a PIXcel detector and fixed slits, using Fe-filtered Co-Kα radiation. The resulting phases were identified through X’Pert Highscore Plus software, and their relative phase amounts (weight %) were estimated using the Rietveld refinement method. The analysis revealed that the recovered Na_2_SO_4_∙10H_2_O had a purity of 84.9% (Fig. 5), confirming the capability of the process to produce high-quality salt suitable for industrial applications while highlighting the need for further optimisation to achieve higher purity levels.

#### Ice Recovery and Energy Usage

**Fig. 6:**
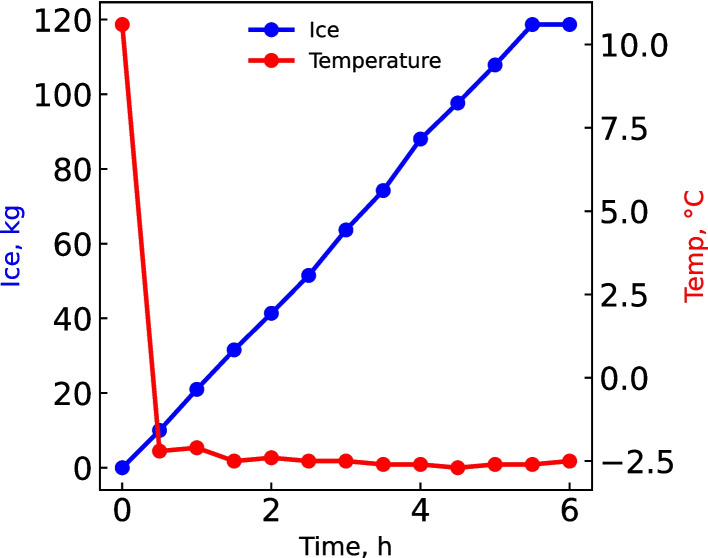
Ice production during cooling of the leachate

Ice production was monitored over time when 273 L of leachate was recycled. This resulted in a mass of 118.7 kg ice being removed over a period of 5.5 h (Fig. 6). The ice purity amounted to 4.0 g L^−1^ TDS, which is significantly lower than the 46.0 g L^−1^ TDS of the feed or 65.9 g L^−1^ TDS of the brine (Table 5). During this period, the energy consumption amounted to 171 kWh t^−1^ ice, which compares well with the theoretical value of 91 kWh t^−1^ ice for a coefficient of performance of 1 (Table SM4).

**Table 5:** Energy usage and comparison of the chemical composition for the leachate, brine and ice

Parameter	Leachate	Brine	Ice		
Time, h	0.0	5.5	1.5	2.5	5.5
Feed, L	273	273	305	325	392
Ice removed, kg	–	–	31.6	51.5	119
New leachate, kg	–	–	31.6	51.5	119
pH	8.6	8.9	9.1	9.2	9.1
COD, mg L^−1^ O_2_	9 150	1 590	588	659	889
Cl^−^, mg L^−1^	8 000	13 900	2 080	1 700	1 400
F^−^, mg L^−1^	70.0	120	0.1	0.1	0.1
NO_3_^−^, mg L^−1^ N	0.0	0.0	0.0	0.0	0.0
SO_4_^2−^, mg L^−1^	16 000	27 900	258.0	391.0	553.0
Na, mg L^−1^	12 500	21 800	1 153	787.0	1 100
K, mg L^−1^	100	170	36.8	53.4	80.6
Mg, mg L^−1^	174	302	7.20	10.0	13.3
Ca, mg L^−1^	61	110	9.1	9.3	10.4
TDS, g L^−1^	50.05	65.89	4.132	3.610	4.046
Cations, meq L^−1^	563	983	52.1	36.9	51.5
Anions, meq L^−1^	562	979	64.0	56.0	51.0
Ice, kg h^−1^	–	–	21.0	10.0	13.4
Energy, kWh	–	–	3.6	7.0	18.6
Energy usage, kWh t^−1^ ice	–	–	171	171	173

The demonstration plant successfully produced 118.7 kg of ice with a notably low TDS concentration, highlighting the capability of the process to generate high purity ice. Energy consumption recorded, though higher than the theoretical minimum, remained within an acceptable range, demonstrating that the process can be scaled up without high energy costs. The results highlight the practical feasibility of freeze crystallization for efficient leachate treatment, combining effective ice recovery with manageable energy demands.

**Fig. 7:**
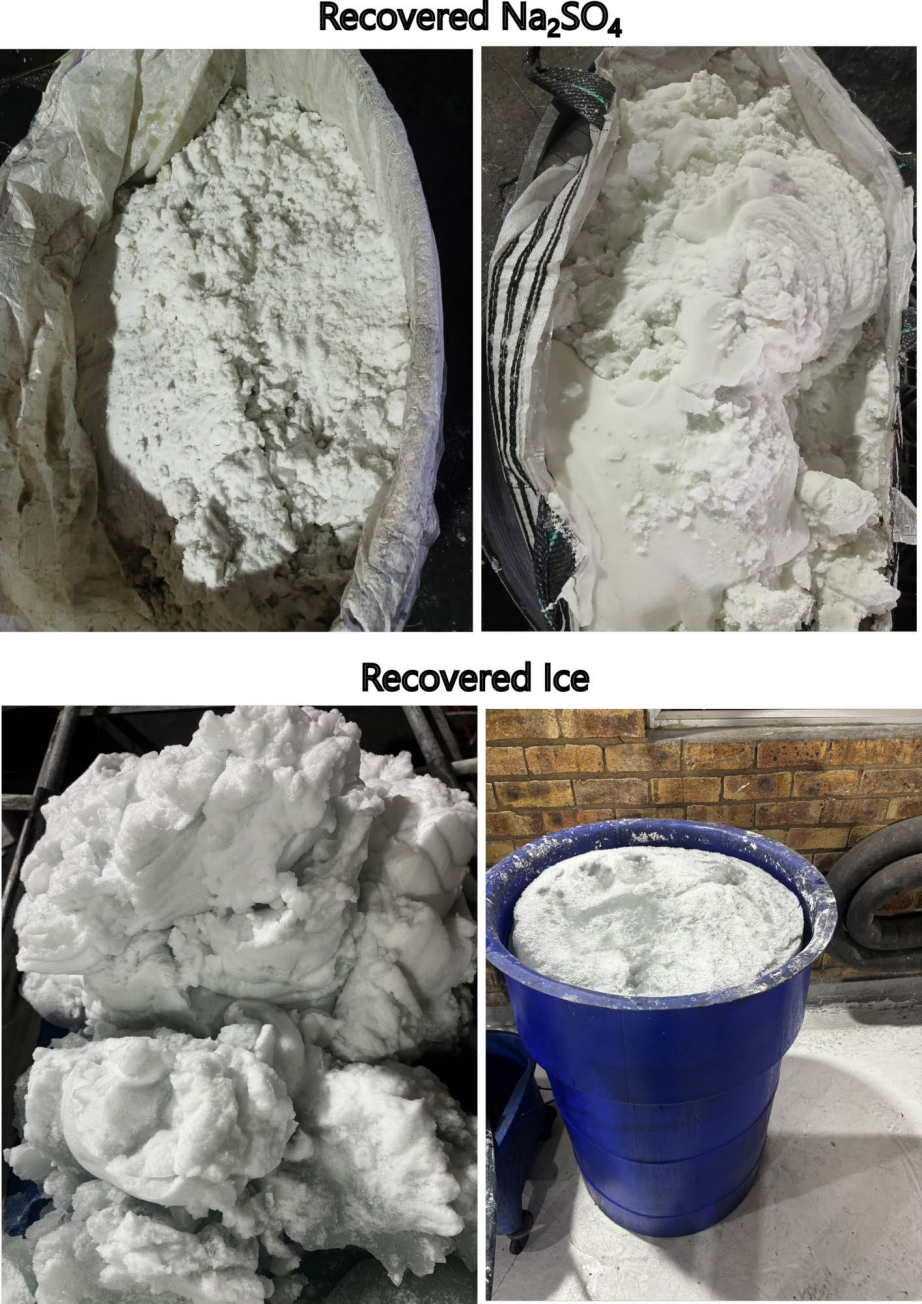
Samples of recovered ice and Na_2_SO_4_

Ice was formed while 273 L of leachate was recycled between the heat exchange and the clarifier. Ice crystals floated in the clarifier and was transferred manually to the ice filter, where ice was recovered. The filtrate was collected in 20 L buckets and returned to the clarifier. Freeze crystallization technology proved to be effective when both the concentrate and leachate were recycled and ice and Na_2_SO_4_ were recovered in large quantities (Fig. 7).

### Evaluation of Freeze Crystallization Efficacy

The freeze crystallization process effectively recovered both salts and ice from hazardous leachate, proving its efficacy as a treatment method. The ability of the process to recover good amount of Na_2_SO_4_.10H_2_O, with actual recovery closely matching predicted values, highlights its precision and reliability. Recovery of high purity ice, with lower TDS concentrations compared to the feed and brine, further highlights the capability of the process to remove contaminants from water, ensuring that the recovered water is of a much higher quality.

Energy consumption data further showcase the efficacy of freeze crystallization, which is competitive and feasible for industrial scale operations. The recorded energy usage for both salt and ice recovery aligns with the intended efficiency of the process, demonstrating that freeze crystallization can be implemented without incurring excessive energy costs. In addition, the flexibility of the process in handling varying operational conditions, as noticed in the different cooling setups and recycling stages, points to its adaptability in real world applications.

The economic evaluation of PFC in comparison with classical leachate treatment methods (evaporative-based methods) demonstrates its cost-effectiveness and operational advantages. Unlike distillation, which has significantly higher energy demands, and evaporation ponds, which require large land areas and pose environmental risks, PFC offers a scalable and environmentally sustainable solution. The operational cost for freeze crystallization, calculated at $2.5 m^−3^, is considerably lower than distillation ($4.5 m^−3^) and marginally higher than evaporation ponds ($2 m^−3^) (Table 6). However, PFC uniquely recovers valuable resources such as Na_2_SO_4_ and clean water, offsetting costs through resource recovery and supporting circular economy initiatives. Na_2_SO_4_ is economically valuable, as it can be sold to paper and glass manufacturing industries. In addition, through thermal treatment, Na_2_SO_4_ can be converted to Na_2_CO_3_ which is more economically valuable as it can be used in the manufacturing of detergents. These findings align with studies by Randall and Nathoo (2015), who also highlighted the energy and operational efficiency of freeze crystallization (Eutectic Freeze Crystallization) for industrial brine management, emphasising its adaptability and scalability for hazardous waste applications.

**Table 6:** Comparative economics of leachate treatment methods

Parameter	Pipe Freeze Crystallization	Distillation	Evaporation Ponds
Capital cost for 0.1 m^3^ h^−1^ plant, $	~ 89,330	~ 134,000	~ 70,000
Operational cost, $ m^−3^	~ 15	~ 40	~ 8
Energy use, kJ kg^−1^	330	2,200	Solar energy
Resource recovery	Na_2_SO_4_, NaCl, clean water	Water	None
Environmental risks	Low	Moderate (High CO_2_)	High (soil or groundwater contamination)
Ease of application	High (compact system)	Moderate (energy dependent)	Low (large land requirement)
Scalability	High	Moderate	Low
CO_2_ emissions, kg CO_2_ t^−1^	134	740	Variable
Best use case	Hazardous and non-hazardous leachate, acid mine drainage, highly saline water, RO brine	High purity water	Low-Tech, arid regions

## Plant Design

### Process Configuration and Calculations

**Fig. 8:**
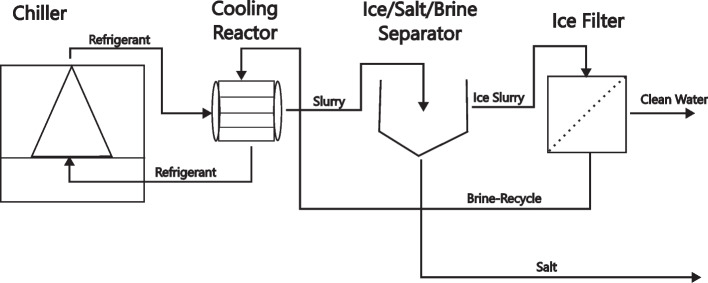
Process configuration for freeze crystallization system

The PFC process implemented in this study consists of a chiller, cooling reactor, ice/salt/brine separator and ice filter with 1 mm pore spaces (Fig. 8). This process begins with the feedwater being introduced through a 32 mm outer diameter heat-exchanger pipe, which extends 150 m in length. The feedwater is maintained at a flow rate that ensures the water is cooled to its freezing point or just below it. This design allows for rapid cooling due to the substantial temperature gradient between the brine and the refrigerant within the chiller system. Once the water is cooled, it is cycled between the cooling reactor and the salt/ice/brine separator. This cycling facilitates the formation of salts and ice. In cases where ice formation is the primary goal, ice is removed from the top layer of the salt/ice/brine separator. To optimise ice production, water treated for salt removal at approximately –9°C serves as the feedstock for the ice production stage.

This study’s objective was to establish design parameters for a freeze crystallization plant capable of producing 100 kg of ice per hour, equivalent to 2.4 t per day. According to the results obtained, a chiller with a cooling capacity of 11.07 kW is required to reduce the temperature of 100 L h^−1^ of feedwater from 25 °C to –21 °C and subsequently freeze it. The calculations further indicate that a pipe length of 118 m is necessary for a PEX pipe with an inner diameter of 55 mm to achieve the required cooling. In addition, a residence time of 34.6 min is important for the complete freezing of the water. If the objective is only to cool the water to its freezing point without complete freezing, the required cooling capacity would be reduced to 3.6 kW. This process configuration and the associated calculations shows the efficiency and scalability of the freeze crystallization method, providing a clear design and implementation of a full scale plant. This approach not only ensures the effective separation and recovery of salts and ice but also optimises energy usage, making the process both economically viable and environmentally sustainable for industrial applications.

### Cost Analysis and Economic Viability of Pipe Freeze Crystallization

The economic viability of the freeze crystallization process is evaluated through a detailed cost analysis, considering both capital and operational expenses. The total costs associated with the transportation and disposal of waste are important, amounting to ZAR2,500 per m^3^ (138.80 $ m^−3^). This highlights the high costs of conventional waste disposal methods. The capital investment required for a freeze crystallization plant with a capacity of 0.1 m^3^ h^−1^ is substantial, estimated at ZAR1,608,000 (89,330 $ m^−3^). When calculated over 120 months at an interest rate of 5% per annum, the capital redemption cost alone amounts to ZAR233.76 per m^3^ (12.95 $ m^−3^). When combined with other operational costs, including electricity, labour, maintenance and chemicals, the total running cost of the freeze crystallization process reaches ZAR524.07 per m^3^ (29.10 $ m^−3^) (Table 7). In addition, there is potential for cost reduction through the processing and sale of by-products. For example, Na_2_SO_4_.10H_2_O can be further processed into Na_2_CO_3_, which has a much higher market price, ZAR10,000 per t (555.50 $ t^−1^) compared to ZAR700 per t (38.85 $ t^−1^) for Na_2_SO_4_. Similarly, valuable products like Al_2_(SO_4_)_3_ and MgSO_4_ can be recovered from specific waste streams, adding further economic value.

**Table 7:** Feasibility of the freeze crystallization plant for leachate treatment

Cost item	Unit	Amount
Capital cost for 0.1 m^3^ h^−1^ plant	ZAR ($)	1,608,000 (89,330)
Capital redemption cost (5%/year, 120 months)	ZAR m^−3^ ($ m^−3^)	233.76 (12.95)
Electricity (125.7 kWt/ice, ZAR1.50/kWh)	ZAR m^−3^ ($ m^−3^)	188.55 (10.40)
Labour	ZAR m^−3^ ($ m^−3^)	50.00 (2.75)
Maintenance (20% of capital redemption cost)	ZAR m^−3^ ($ m^−3^)	46.75 (2.55)
Chemicals	ZAR m^−3^ ($ m^−3^)	5.00 (0.25)
Total cost of Freeze Crystallization	ZAR m^−3^ ($ m^−3^)	524.07 (29.10)
Disposal cost at toxic waste disposal sites	ZAR m^−3^ ($ m^−3^)	2,500.00 (138.80)

Scaling up the PFC process for application in a real landfill is both feasible and practical, particularly when compared to existing evaporative-based techniques such as distillation and evaporation ponds. These conventional methods dominate brine treatment due to their simplicity and ability to concentrate large volumes of waste. However, they come with significant disadvantages as distillation is highly energy-intensive, leading to extremely high operational costs, especially at larger scales. Evaporation ponds, while low-cost in terms of operation, require large amounts of land, making them unsuitable for urban areas or regions with land constraints. Furthermore, evaporation ponds pose severe environmental risks, as metals and salts from the brine can seep into the ground, contaminating soil and groundwater over time. In contrast, PFC requires significantly less energy, and does not depend on large land areas or pose similar environmental risks, making it a more sustainable and scalable alternative.

The scalability of PFC is already demonstrated by its application in a pyrolysis plant in Midrand, Gauteng province of South Africa, where it successfully treats 300 L h^−1^ of highly saline wastewater. This operational setup has proven its ability to handle industrial effluent effectively, showcasing the flexibility of the process in managing varying wastewater compositions. The modular nature of PFC plants allows for capacity adjustments by incorporating additional reactors and clarifiers to meet higher treatment demands. Furthermore, the process is designed to recover valuable by-products such as Na_2_SO_4_, NaCl and clean water, which can offset operating costs and generate revenue, enhancing economic feasibility. These advantages position PFC as a viable, scalable solution for hazardous landfill leachate treatment, offering a cost-effective and environmentally friendly alternative to traditional methods.

## Limitations of the Study

One notable limitation of the study is the absence of long-term operational data on the type of leachate examined in this study to evaluate the durability and reliability of the PFC system. While the demonstration plant achieved good results in recovering salts and ice, the short-term nature of the experiments does not provide sufficient insights into the durability of system components, potential fouling or scaling issues, and maintenance requirements over extended periods. This limitation raises concerns about the technology’s robustness and operational consistency in real-world applications, where continuous operation under varying conditions is required. Future studies will include prolonged trials to assess these aspects and validate the system's reliability for industrial application.

Another limitation is related to the variability in leachate composition. While the study demonstrated the efficacy of PFC for a specific leachate type with known concentrations of TDS and chloride, the results may not directly translate to leachates with significantly different characteristics. Factors such as the presence of organic contaminants, metals, or other impurities could affect the crystallization process, reducing efficiency or product purity. The lack of a comprehensive analysis on how diverse feedwater qualities affect system performance leaves a gap that must be addressed to improve the applicability of the technology across different industries and regions.

It is also worth noting that the economic analysis, while robust, relies heavily on assumptions regarding operational conditions, market value of recovered salts, and cost stability. Variables such as energy prices, equipment maintenance costs, and demand for by-products like Na_2_SO_4_ and NaCl may fluctuate, affecting the long-term viability of the process. In addition, the study focuses primarily on direct operational costs and benefits, without a detailed life cycle assessment to evaluate the broader environmental and economic impacts, such as carbon offsets or reduced landfill usage. These limitations highlight the need for further research to refine the technology and provide more comprehensive data to support its adoption on a global scale. As this is a new and developing technology, further research will still be conducted, and more data will be gathered to support the technology.

## Conclusions and Recommendations

This study demonstrates the efficacy of PFC as a sustainable and innovative approach for managing hazardous landfill leachate, focusing on the recovery of valuable resources like Na_2_SO_4_∙10H_2_O and clean water. Unlike traditional evaporative methods, PFC successfully integrates resource recovery with environmental compliance, presenting a scalable solution for leachate treatment. The demonstration plant results validated the technology, with a recovery of 102.9 kg of Na_2_SO_4_∙10H_2_O with 84.9% purity and 118.7 kg of ice, closely matching predictive outputs from OLI simulations. These findings highlight the precision of the process design and its ability to achieve high recovery rates with efficient energy usage. In addition, the study highlights the broader economic viability of the technology, emphasising the potential to convert recovered products into higher-value derivatives, such as Na_2_CO_3_, enhancing its market appeal.

The advantages of PFC over traditional methods, such as reduced energy consumption and the elimination of environmental risks associated with evaporation ponds, make it a good alternative. Furthermore, its ability to reduce TDS and Cl^−^ concentrations in effluents aligns with the goals of zero liquid discharge, ensuring compliance with increasingly stringent environmental regulations. The system’s modular and flexible design also allows for potential adaptation to a variety of waste streams beyond leachate, such as mining and industrial effluents, broadening its applicability.

Future work will prioritise exploring alternative refrigerants with lower global warming potential to enhance energy efficiency further. Developing a comprehensive economic model to optimise operational costs and maximise revenue through product recovery will also be critical. The inclusion of real-time monitoring and control systems, such as a Python-based SCADA system, could improve operational precision and scalability for industrial-scale applications. This study also recommends the construction of a full-scale PFC plant at the landfill site studied, providing a practical demonstration of its cost-effectiveness and environmental benefits while setting a benchmark for future applications in the waste management industry.

## Supplementary Information

Below is the link to the electronic supplementary material.

Supplementary file1 (DOCX 52 KB)

## Data Availability

The data and materials are not publicly available due to privacy/ethical restrictions.
