# Supplementary material for: Cost-Effective Leachate Treatment and Resource Recovery in Hazardous Waste Landfills through Pipe Freeze Crystallization
Source: Environ Process. 2025 Mar 24;12(2):15. doi: 10.1007/s40710-025-00757-3 (PMC11956536; doi:10.1007/s40710-025-00757-3)
Supplement: Supplementary file 1 — Supplementary file1 (DOCX 52 KB) [file 40710_2025_757_MOESM1_ESM.docx]

SUPPLEMENTARY MATERIAL

Cost-Effective Leachate Treatment and Resource Recovery in Hazardous Waste Landfills through Pipe Freeze Crystallization

**Kagiso S. More^*^, Johannes P. Maree and Mlungisi Mahlangu**

*Institute for Nanotechnology and Water Sustainability, College of Science, Engineering and Technology, University of South Africa, Private Bag X6, Science Campus, Florida, Johannesburg, 1709, South Africa*

^*^ Corresponding author: Tel.: +27 736084241; E-mail: moreks@unisa.ac.za

Kagiso S. More ORCID 0000-0003-2803-7983 [moreks@unisa.ac.za](mailto:moreks@unisa.ac.za)

Johannes P. Maree ORCID 0000-0002-8901-2512 mareejp@unisa.ac.za

Mlungisi Mahlangu ORCID 0000-0002-0270-8227 11968680@mylife.unisa.ac.za

**Table SM1**. Full chemical composition of samples collected at the waste management company

| **Parameter** | **Unit** | **Sample A: Evaporation Concentrate** | **Sample B: Leachate** |
| --- | --- | --- | --- |
| pH |  | 6.69 | 8.64 |
| COD | mg L^-1^ O_2_ | 39,900 | 10,400 |
| Total Organic Carbon (OL) | mg L^-1^ | 10,600 | 4,820 |
| Total Cyanide in liquid | mg L^-1^ CN | 0.05 | 0.32 |
| Total Phenols in liquid | mg L^-1^ Phenol | 0.13 | 1.03 |
| Total Oil and Grease | mg L^-1^ | 188 | 0 |
| Anionic surfactants | mg L^-1^L | 24 | 5 |
| Formaldehyde | µg L^-1^ | 727 | 884 |
| Free Chlorine | mg L^-1^ | 0 | 0 |
| Total Conductivity | mS m^-1^ | 11,900 | 3,810 |
| P Alkalinity (to pH 8.3) | mg L^-1^ CaCO_3_ | 0 | 1,220 |
| Total (M) Alkalinity (to pH 4.3) | mg L^-1^ CaCO_3_ | 5,060 | 19,300 |
| Cl^-^ | mg L^-1^ | 25,953 | 8,238 |
| F^-^ | mg L^-1^ | 133.00 | 83.40 |
| NO_3_^-^ | mg L^-1^ | 55.60 | 6.27 |
| NO_3_ as N | mg L^-1^ | 12.60 | 1.42 |
| PO_4_^3-^ as P | mg L^-1^ | 47.51 | 22.34 |
| SO_4_^2-^ | mg L^-1^ | 121,700 | 16,330 |
| Sulphides | mg L^-1^ | 13.7 | 860 |
| Ammonia as N | mg L^-1^ | 972 | 252 |
| Na | mg L^-1^ | 62,464 | 23,765 |
| K | mg L^-1^ | 7,690 | 22 |
| Mg | mg L^-1^ | 386 | 191 |
| Ca | mg L^-1^ | 124 | 57 |
| Al | mg L^-1^ | 2.99 | 0.48 |
| As | mg L^-1^ | 2.99 | 3.14 |
| B | mg L^-1^ | 31.50 | 13.10 |
| Cd | mg L^-1^ | 0.00 | 0.02 |
| Co | mg L^-1^ | 0.27 | 0.14 |
| Cr | mg L^-1^ | 11.80 | 6.57 |
| Cu | mg L^-1^ | 0.00 | 0.01 |
| Fe | mg L^-1^ | 11.80 | 3.14 |
| Mn | mg L^-1^ | 7.84 | 2.19 |
| Mo | mg L^-1^ | 1.75 | 1.47 |
| Ni | mg L^-1^ | 2.42 | 1.68 |
| Pb | mg L^-1^ | 0.00 | 0.02 |
| Se | mg L^-1^ | 0.23 | 0.74 |
| Ti | mg L^-1^ | 2.31 | 1.76 |
| Zn | mg L^-1^ | 0.30 | 0.00 |
| Ag | mg L^-1^ | 1.66 | 0.02 |
| Hg | mg L^-1^ | 0.08 | 0.04 |
| W | mg L^-1^ | 2.66 | 0.57 |
| Anions | meq L^-1^ | 3,385 | 1,019 |
| Cations | meq L^-1^ | 3,016 | 1,068 |
| Total dissolved solids | mg L^-1^ | 220,201 | 50,005 |
| Suspended Solids at 105°C | mg L^-1^ | 770 | 20 |

**Table SM2**. Full water quality results through various stages of freeze crystallization predicted using OLI software; sample: concentrate

| **Parameter** | **Unit** | **Feed** | **Cooling** | **Freeze 1** | **Freeze 2** | **Freeze 3** |
| --- | --- | --- | --- | --- | --- | --- |
| H_2_O | kg h^-1^ | 1000 | 739.28 | 116.28 | 22.70 | 11.99 |
| Ice recovery | % |  |  | 64.6 | 9.4 | 1.1 |
| Temperature | °C | 25.00 | 0 | -4 | -21 | -21 |
| pH |  | 7.57 | 7.93 | 7.22 | 6.67 | 6.71 |
| **Solids:** | | | | | | |
| Ice |  |  |  | 645.94 | 93.58 | 10.71 |
| Na_2_SO_4_.10H_2_O (Mirabilite) | kg h^-1^ | 0.00 | 353.88 | 41.04 | 0.00 | 0.00 |
| Ca_5_F(PO_4_)_3_ (Fluorapatite) | kg h^-1^ | 0.26 | 0.26 | 0.00 |  | 0.00 |
| NaMgF_3_ (Neighborite) | kg h^-1^ | 0.17 | 0.17 | 0.03 | 0.00 | 0.00 |
| CaF_2_ (Fluorite) | kg h^-1^ | 0.00 | 0.03 |  |  | 0.00 |
| NaHCO_3_ (Nahcolite) | kg h^-1^ |  |  | 6.28 | 1.32 | 0.21 |
| KCl (sylvite) | kg h^-1^ |  |  | 0.24 | 9.92 | 0.33 |
| CaCO_3_ (Calcite) | kg h^-1^ |  |  | 0.01 |  | 0.00 |
| NaCl (Halite) | kg h^-1^ |  |  | 0.00 | 25.23 | 2.94 |
| K_2_SO_4_.KNaSO_4_ (Aphthitalite) | kg h^-1^ |  |  |  | 3.28 | 0.51 |
| Na_2_O.5B_2_O_3_.2H_2_O | kg h^-1^ |  |  |  | 0.07 | 0.04 |
| K_2_SO_4_.CaSO_4_.1H_2_O (Syngenite) | mg L^-1^ |  |  |  | 0.01 | 0.00 |
| K_2_SO_4_.MgSO_4_.6H_2_O (Schoenite) | kg h^-1^ |  |  |  | 0.00 | 1.23 |
| NH_4_Cl (Sal ammoniac) | kg h^-1^ |  |  |  | 0.00 | 1.16 |
| Mass - Solid | kg h^-1^ | 0.42 | 354.33 | 47.61 | 39.85 | 6.42 |
| Mass - Solid | % | 0.09 | 78.98 | 10.61 | 8.88 | 1.43 |
| **Solution:** | | | | | | |
| SO_4_ | kg h^-1^ | 121.87 | 16.21 | 3.96 | 2.05 | 1.16 |
| Cl^-^ | kg h^-1^ | 25.95 | 25.95 | 25.84 | 5.33 | 3.38 |
| F | kg h^-1^ | 0.03 | 0.02 | 0.00 |  |  |
| C | kg h^-1^ | 1.18 | 1.18 |  |  |  |
| Na | kg h^-1^ | 70.94 | 20.44 | 12.85 | 2.33 | 1.08 |
| K | kg h^-1^ | 7.69 | 7.69 | 7.56 | 1.20 | 0.61 |
| NH_4_-N | kg h^-1^ | 0.75 | 0.75 | 0.75 | 0.75 | 0.45 |
| Mg | kg h^-1^ | 0.35 | 0.35 | 0.34 | 0.34 | 0.27 |
| Ca | kg h^-1^ | 0.02 | 0.01 | 0.00 |  |  |
| B | kg h^-1^ | 0.03 | 0.03 |  |  |  |
| P | kg h^-1^ | 0.00 | 0.00 | 0.00 | 0.00 | 0.00 |
| TDS | kg h^-1^ | 228.82 | 72.63 | 51.32 | 12.01 | 6.94 |
| Mass - Liquid-1 | g L^-1^ | 1,170.95 | 817.04 |  |  |  |
| Cations | meq L^-1^ | 3.37 | 1.17 | 0.83 | 0.21 | 0.12 |
| Anions | meq L^-1^ | 3.37 | 1.17 | 0.81 | 0.19 | 0.12 |
| Energy (cooling) | J L^-1^ Feed |  | 6,180 | 9,235 |  |  |
| Energy (freezing) | J L^-1^ Feed |  |  |  | 31,163 | 3,566 |
| Total energy | J L^-1^ Feed |  | 50,145 | | | |
| T1 | °C |  | 0 | -2 |  |  |
| T2 | °C |  | -2 | -21 | -21 | -21 |

**Table SM3**. Full water quality results through various stages of freeze crystallization predicted using OLI software; sample: leachate

| **Parameter** | **Unit** | **Feed** | **Cool** | **Na_2_SO_4_** | **NaCl** |
| --- | --- | --- | --- | --- | --- |
| H_2_O | kg h^-1^ | 982.10 | 957.82 | 101.31 | 6.00 |
| pH |  |  |  | 7.50 | 6.71 |
| Temperature | °C | 25 | 0 | -4 | -21 |
| Ice | kg h^-1^ | 0.00 | 0.00 | 856.51 | 95.31 |
| Na_2_SO_4_·10H_2_O (Mirabilite) | kg h^-1^ | 0.00 | 43.42 | 71.99 | 1.02 |
| NaHCO_3_ (Nahcolite) - Sol | kg h^-1^ |  |  | 9.71 | 1.12 |
| NaMgF_3_ (Neighborite) - Sol | kg h^-1^ | 0.05 | 0.06 | 0.07 | 0.00 |
| Ca_5_F(PO_4_)_3_ (Fluorapatite) - Sol | kg h^-1^ | 0.12 | 0.12 | 0.00 |  |
| CaF_2_ (Fluorite) - Sol | kg h^-1^ | 0.00 | 0.01 |  | 9.48 |
| Na_2_O·5B_2_O_3_·2H_2_O | kg h^-1^ |  |  |  | 0.04 |
| NH_4_Cl (Sal ammoniac) - Sol | kg h^-1^ |  |  |  | 0.05 |
| CaCO_3_ (Calcite) - Sol | kg h^-1^ |  |  | 0.01 | 0.00 |
| Ca_5_F(PO_4_)_3_ (Fluorapatite) - Sol | kg h^-1^ |  |  | 0.00 | 0.00 |
| **Solution:** | | | | | |
| Na^+1^ | kg h^-1^ | 23.75 | 17.56 | 4.61 | 0.43 |
| K^+1^ | kg h^-1^ | 0.02 | 0.02 | 0.02 | 0.02 |
| NH_4_^+^ | kg h^-1^ | 0.20 | 0.20 | 0.20 | 0.18 |
| Mg^+2^ | kg h^-1^ | 0.18 | 0.18 | 0.16 | 0.16 |
| Ca^+2^ | kg h^-1^ | 0.01 | 0.00 | 0.00 | 0.00 |
| SO_4_^-2^ | kg h^-1^ | 35.19 | 22.23 | 0.74 | 0.14 |
| Cl^-1^ | kg h^-1^ | 7.00 | 7.00 | 7.00 | 1.22 |
| F^-1^ | kg h^-1^ | 0.05 | 0.04 | 0.00 | 0.00 |
| C^+4^ | kg h^-1^ | 1.57 | 1.57 | 0.19 | 0.02 |
| P^+5^ | kg h^-1^ | 0.00 | 0.00 | 0.00 | 0.00 |
| B^+3^ | kg h^-1^ | 0.01 | 0.01 | 0.01 | 0.00 |
| TDS | kg h^-1^ | 67.99 | 48.81 | 12.93 | 2.18 |

**Table SM4**. Energy usage for ice production when coefficient of production is 1

| **Parameter** | **Unit** | **Value** |
| --- | --- | --- |
| Flow | t h^-1^ | 1.00 |
| Hv | kJ kg^-1^ | 330.00 |
| Energy | kJ h^-1^ | 330,000.00 |
| Time | sec h^-1^ | 3,600.00 |
| Energy | kW | 91.67 |
| Energy utilization | kWh t^-1^ | 91.67 |
